# What Is the Survivorship of Megaprosthetic Reconstruction Following the Resection of Renal Cell Carcinoma Long Bone Metastases and What Are the Potential Risk Factors for a Prosthetic Complication? †

**DOI:** 10.3390/cancers17121982

**Published:** 2025-06-13

**Authors:** Sebastian Bockholt, Kristian Nikolaus Schneider, Georg Gosheger, Maria Anna Smolle, Niklas Deventer, Dimosthenis Andreou, Christoph Theil

**Affiliations:** 1 Department of Orthopedics and Tumor Orthopedics, Muenster University Hospital, Albert-Schweitzer-Campus 1, 48149 Muenster, Germany; sebastian.bockholt@ukmuenster.de (S.B.); kristian.schneider@ukmuenster.de (K.N.S.); georg.gosheger@ukmuenster.de (G.G.); niklas.deventer@ukmuenster.de (N.D.); dimosthenis.andreou@uk-essen.de (D.A.); 2 Department of Orthopaedics and Traumatology, Medical University of Graz, Auenbruggerplatz 5, 8036 Graz, Austria; maria.smolle@medunigraz.at; 3 Institute for Interdisciplinary Sarcoma Treatment and Research, Department of Orthopaedic Oncology and Sarcoma Surgery, University Hospital Essen, Hufelandstraße 55, 45147 Essen, Germany

**Keywords:** bone metastasis, megaprosthesis, megaprostheses, renal cell caricnoma

## Abstract

**Background:** Long bone metastases are common in patients with metastatic renal cell carcinoma (RCC). One potential surgical treatment option is resection and megaprosthetic reconstruction. However, implant complications and survival are poorly understood. This study analyzes patient and implant survival as well as associated risk factors. **Methods:** This is a retrospective study from a single academic center, analyzing 86 patients that underwent resection and megaprosthetic reconstruction performed between 1993 and 2017. The most common location of megaprosthetic reconstruction was the proximal femur (PFR) in 38% (33 of 86) of patients. We calculated overall patient survival and associated risk factors using the Kaplan–Meier method and implant survivorship using a competing risk analysis. **Results:** A total of 73% (63/86) of patients died of their disease after a median of 19 (IQR 9–37) months following surgery, and a median of 71 (IQR 31–132) months after the initial diagnosis of RCC. The overall survival probability was 29% (95% CI 18–40%) five years after surgery. The five-year risk of revision surgery (within a competing risk framework) was 18% (95% CI 11–28). A total of 8% (7 of 86) of patients underwent an exchange of the implant itself. Patients with total bone replacements had a higher revision risk (SHR 19.46 (95% CI 6.9–54.9), *p* < 0.01). Furthermore, the revision risk was higher with increasing reconstruction length per mm (SHR 1.01 (95% CI 1.01–1.02), *p* = 0.03) and prolonged surgical time per minute (SHR: 1.01 (95% CI 1.0–1.02), *p* < 0.01). Local postoperative radiation treatment (RTX) was associated with an increased risk for revisions (SHR 2.59 (95% CI 0.96–6.95), *p* = 0.06). **Conclusions:** Modular megaprostheses demonstrated a fairly low risk of implant revision although postoperative radiation therapy and total bone replacements are associated with an increased risk.

## Simple Summary

Kidney cancer can also spread to the long bones and cause pain, instability or even fractures. In these situations surgery is an option. One type of surgery is to remove the part of the bone that is affected by the tumor and replace the defect with a metal prosthesis. Considering the potential impact on the affected patients, a further understanding of the associated complications and implant survival is needed. This study investigated 86 patients who were treated in this fashion. It was found that the long-term risk for complications was 18% and that soft tissue complications were the most common type of failure. Larger implants and particularly those that replaced entire bones were associated with a greater risk of complications. Nonetheless, considering the decent survival of patients with this type of cancer, the risk of complications is fairly low.

## 1. Introduction

Bone metastases are frequent in patients with metastatic renal cell carcinoma (RCC), occurring in up to 49% of cases [1,2]. Considering a reported overall survival of approximately 33% at five years, particularly following the introduction of novel systemic treatment options, and the relative resistance of RCC metastases to radiation therapy, local surgical therapy is of great importance, especially in patients presenting with impending or actual pathological fractures [1,2,3,4,5,6]. While in the past bone metastases only required temporary stabilization [7,8] for painful lesions or following pathological fracture given the short life expectancy of patients with metastasized disease, long-lasting reconstructive surgery has become more common [4,9,10,11,12,13]. Furthermore, in recent years, the complete en bloc metastasectomy of solitary and oligometastases of RCC has been associated with a longer overall survival probability [4,14,15]. One study investigated 101 patients who underwent surgery for RCC metastases, of whom 37 had prosthetic reconstruction. They found that complications occurred in 9% of surgeries; however, there was no stratification regarding the type of surgery and it is unclear how many patients underwent megaprosthetic reconstruction. Considering the poor response to radiation as well as the reasonable overall survival, they argued that resection with tumor-free margins and reconstruction is a reasonable approach for solitary or oligometastatic lesion [15].

After long bone metastasectomy, megaprosthetic reconstruction is frequently employed in the management of such defects [16,17,18]. The main concern in the use of these implants is the fairly high revision risk which needs to be balanced with surgical morbidity in patients with limited life expectancy [19]. Implant survival, complications, and risk factors for the implant revision of megaprosthetic reconstruction for RCC metastases are widely unclear, with only a few studies having evaluated this issue thus far [14,20,21]. Furthermore, previous studies have relied on Kaplan–Meier analysis of implant survivorship [14,18,20,22], being prone to overestimation of implant-related complications when an obvious competing risk like patient death is present [23]. Therefore, it is unclear to what extent implant complications occur in this specific patient cohort and how this influence surgical decision making.

The aim of the study was to investigate the implant survival of megaprosthetic reconstruction in patients with long bone metastases of RCC and specifically answer the following questions:

1. What is the overall survival of patients with RCC bone metastases treated with megaprosthetic reconstruction, and which factors influence survival?
2. What is the revision-free implant survivorship of modular megaprostheses used in the reconstruction of bone defects following the resection of a RCC metastasis in a competing risk framework, and what are types and timing of implant complications?
3. Which factors are associated with a risk of implant complications?

## 2. Patients and Methods

### 2.1. Study Design and Setting

In this retrospective study, we investigated patients who underwent upper and lower limb megaprosthetic reconstruction for long bone metastasis at a single academic, tertiary referral center for orthopedic oncology using a single modular implant system (MUTARS-modular universal tumor and revision system, Implantcast GmbH, Buxtehude, Germany) between 1993 and 2017.

### 2.2. Patients and Treatment Approach

We identified 234 patients treated during this period. Among those, 86 patients had a metastasis originating from RCC. The indication for resection and prosthetic reconstruction was if the cancer was locally advanced and had caused extensive bone destruction due to a lesion that was not deemed otherwise reconstructable, including impending or actual pathological fractures and patients with failed previous non-megaprosthetic stabilization procedures. Furthermore, resection and megaprosthetic reconstruction was generally recommended in patients with solitary metastases as well as patients with oligometastatic disease who were undergoing further systemic treatment and were fit to undergo major orthopedic surgery. We did not use other implant systems and did not employ other modes of reconstruction in the presence of a segmental bone defect during the study period. All patients underwent routine diagnostics, including a radiographic and magnetic resonance imaging (MRI) of the whole affected bone in order to plan resection. In cases of previously unknown metastatic disease, an open biopsy was performed prior to surgery, as were computed tomography scans of the chest and abdomen for staging purposes. We routinely used a bipolar head for hip joint replacements. Previously implanted acetabular components were retained in patients undergoing revisions when stability was uncompromised; otherwise, a dual mobility cup was implanted. For proximal or total humeral prostheses, an inverse prosthesis was used from 2012 onwards whenever deltoid and preserved axillary nerve function allowed for it.

The decision to perform adjuvant radiation or systemic treatment was based on the recommendations of the local bone and/or urological tumor board. Resection margins were graded as wide, marginal and contaminated based on surgical and pathological reports.

### 2.3. Definitions

Implant complications were classified according to the classification system proposed by Henderson et al. [24], which briefly distinguishes between mechanical (types I–III: soft tissue complication, aseptic loosening, structural complications) and non-mechanical complications (types IV–V: prosthetic infection, tumor recurrence).

### 2.4. Demographic Details

We obtained data from patients’ electronic medical files and recorded complications requiring revision surgery, patient- and procedure-related factors, surgical and oncological details, as well as overall survival (Table 1, Table 2 and Table 3). Follow-up data were derived from the last contact with our institution and/or death of the patient as reported to the state registry.

### 2.5. Ethical Approval

Approval of the institutional ethics committee was obtained prior to this investigation (Ethical Review Board—local ethical committee 2018-601- F-s) and the study was conducted according to the principles of the Declaration of Helsinki.

### 2.6. Statistical Analysis

Data collection and statistical analysis were performed using Excel (Microsoft Corporation, Redmont, Washington, DC, USA), SPSS Version 26 (IBM Corporation, Armonk, NY, USA) and Stata Version 15 (StataCorp LLC, College Station, TX, USA). Descriptive statistics were used to analyze means and standard deviation were calculated for parametric data; medians and interquartile range (IQR) were calculated for non-parametric data. Contingency tables were analyzed using chi^2^-test. Non-parametric analyses were performed using the Mann–Whitney U-test. Patients’ overall survival was calculated with the Kaplan–Meier method [25] and survival curves were compared with the log-rank test [26]. Competing risk regression analyses with death as the competing event were used to estimate implant-specific complication risks [27]. Potential associated risk factors were analyzed with Fine and Gray models reporting subhazard ratios (SHRs) with 95% confidence intervals (95% CI). Univariate Cox regression analyses were performed to assess risk factors affecting patients’ prognosis, considering the limited number of patients and risk factors hampering multivariate analysis. The overall and implant survival probabilities are reported with their respective 95% CIs. All *p*-values were two-sided and statistical significance was defined as *p* ≤ 0.05.

## 3. Results

### 3.1. What Is the Overall Survival of Patients with RCC Bone Metastases Treated with Megaprosthetic Reconstruction and Which Factors Influence Survival?

**Figure 1:**
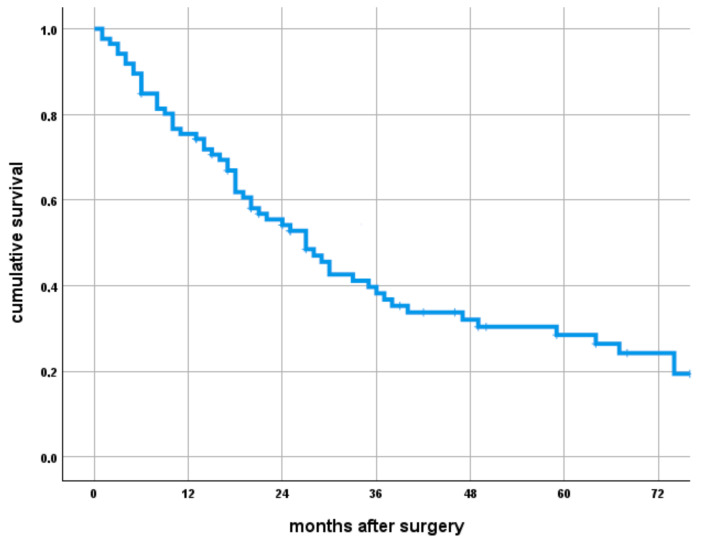
Overall patient survivorship curve in months.

A total of 73% (63/86) of patients died of their disease after a median of 19 (IQR 9–37) months following surgery, and a median of 71 (IQR 31–132) months after the initial diagnosis of RCC. The overall survival probability was 54% (95% CI 43–64%) two years after surgery and 29% (95% CI 18–40%) five years after surgery (Figure 1).

We did not detect a difference in overall survival depending on the timing of the metastases (at initial diagnosis, within two years, later than two years, *p* = 0.37) or with respect to the resection margins (*p* = 0.45). Furthermore, there was no difference in overall survival between patients with a solitary bone metastasis at the time of surgery and patients with multiple metastases (45% (95% CI 34–54) vs. 58% (95% CI 46–60) at two years and 25% (95% CI 9–41) vs. 30% (95 CI 17–43) at five years, *p* = 0.69). In addition, there was no difference in overall survival between patients with a pathologic fracture and patients with impending fractures (50% (95% CI 35–65) vs. 59% (95% CI 43–75) at two years and 26% (95% CI 12–40) vs. 32% (95% CI 16–48), *p* = 0.47).

While patients with additional pulmonary metastases at the time of surgery did not have a reduced overall survival probability five years after surgery (25% (95% CI 10–40%) vs. 31% (95% CI 17–45%), *p* = 0.1), patients with additional visceral metastases had a shorter overall survival (19% (95% CI 9–28%) vs. 31% (19–43%) at five years, *p* = 0.04).

With the numbers available for this analysis, there was no difference in survival at five years between patients who underwent surgery prior to 2006 and later on (25% (95% CI 12–38) vs. 32% (95% CI 14–50), *p* = 0.89).

### 3.2. What Is the Revision-Free Implant Survivorship of Modular Megaprostheses Used in the Reconstruction of Bone Defects Following the Resection of a RCC Metastasis in a Competing Risk Framework, and What Are Types and Timing of Implant Complications?

Nineteen percent (16/86) of patients developed an implant-related complication during follow-up after a median of 3 (IQR 1–15) months. Soft tissue complications were the most common in 12% of patients (10/86), followed by loosening in 3% (3/86), infection in 2% (2/86) and a periprosthetic fracture in 1% (1/86).

**Figure 2:**
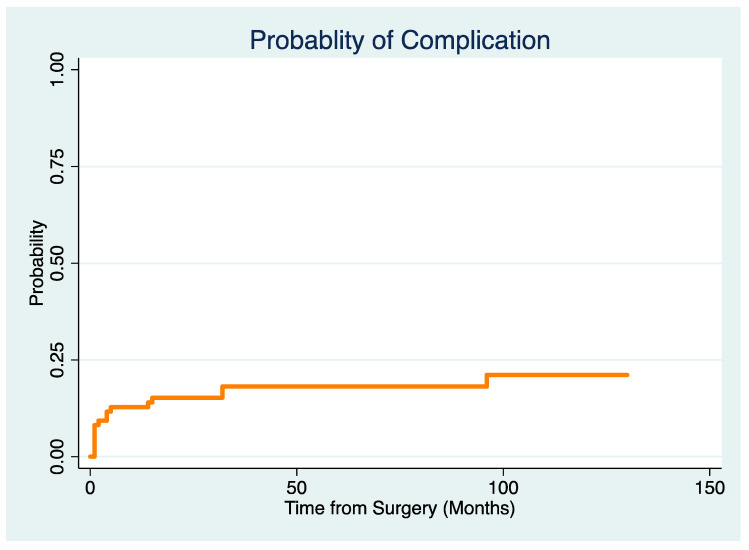
Cumulative incidence function of any implant-related complications over time.

A total of 8% (7/86) of patients underwent a revision of the implant. The two- and five-year implant risk of revision surgery (using a competing risk estimator) was 15% (95% CI –9–24) and 18% (95% CI 11–28) (Figure 2).

### 3.3. Which Factors Are Associated with the Risk for Implant Complications in a Competing Risk Model?

With implants at the proximal end of bones as the reference, patients with total bone implants had a higher risk of developing any implant-related complications (SHR 19.46 (95% CI 6.9–54.9), *p* < 0.01), whilst there was no difference for implants located at the distal ends of bone (SHR 0.53 (95% CI 0.12–2.32), *p* = 0.4) or intercalary implants (SHR: 1.59 (95% CI 0.35–7.32), *p* = 0.55). All patients with a total bone replacement underwent revision surgery. Furthermore, the risk for complications was higher with increasing reconstruction length per mm (SHR 1.01 (95% CI 1.0–1.02), *p* = 0.03) and prolonged surgical time per minute (SHR: 1.01 (95% CI 1.01–1.02), *p* < 0.01).

When investigating the effect of adjuvant treatment, there was an increased risk of complications associated with local postoperative radiation treatment (RTX) (SHR 2.59 (95% CI 0.96–6.95), *p* = 0.06), while on the other hand, preoperative RTX (SHR 1.038 (95% CI 0.34–3.16), *p* = 0.95) or the application of preoperative chemotherapy (CTX) (SHR 0.76 (95% CI 0.22–2.56), *p* = 0.66) or postoperative CTX (SHR 1.37 (95% CI 0.54–3.53), *p* = 0.51) had no impact on the risk for complications. With the numbers we had, we were not able to identify any further risk factors (Table 4).

## 4. Discussion

While bone metastases are common in patients with metastatic RCC, the optimal reconstruction method in patients undergoing surgical treatment is unclear [22,28]. Considering the good overall survival of the affected patients, a long-lasting reconstruction is required, which can be achieved using a modular megaprosthetic implant [20,29]. However, there is a paucity of studies that investigate implant survivorship and the associated risk factors for revision surgery [14] in affected patients and there is no study using a competing risk approach that might result in a more appropriate survival estimate in patients suffering from metastatic disease. Considering that systemic treatment options and the overall prognosis are constantly improving [28], data on implant survivorship are needed to inform clinicians and patients.

The main findings of this study were that there is a satisfactory implant survivorship with a relatively low probability of implant revision. However, total bone reconstructions, postoperative radiation treatment, and the overall length of megaprosthetic reconstruction were associated with a higher probability for implant complications. On the other hand, the presence of visceral metastasis at the time of surgery was the only factor that was found to be associated with a worse overall survival probability. Therefore, resection and megaprosthetic reconstruction appears to be a reliable and safe treatment option for RCC bone metastases. Patients with large lesions that might require (sub-) total bone reconstructions must be counseled regarding the increased risk for complication. Likewise, patients who have large soft tissue masses or pathological fractures with hematoma that would usually require extensive radiation therapy in order to achieve long-term local control must be counseled regarding this risk.

While this is a large single-center study and, as far as we are aware, the first using a competing risk model to investigate implant survivorship after megaprosthetic reconstruction for RCC metastases, it has several limitations: Firstly, it is limited by its retrospective design that entails several inherent limitations and is prone to selection and transfer bias. The latter would be expected to deemphasize the risk of revision surgery as patients might have undergone treatment elsewhere unknown to us and the actual implant survivorship might be lower. However, considering the complexity of these procedures, it appears unlikely that patients are treated at non-specialized centers, limiting this effect. Furthermore, there is a selection bias because we only included megaprosthetic reconstructions. For this procedure, it would be intuitive to assume that patients were deemed to be in fair overall health, considering the risk of a more invasive surgery, and were referred to a specialized tertiary center for orthopedic oncology when megaprosthetic reconstruction was considered by the treating oncologist or an outside orthopedic trauma unit in cases of pathological fractures. Nonetheless, as prosthetic reconstruction is a frequently considered option for metastatic lesions, we believe that the data presented can help in guiding the surgeon’s decision making and patient’s expectations regarding implant survivorship. While we assume that patients were in fair health when resection and reconstruction was performed, pre- and postoperative performance status were not recorded and can therefore not be included in the analysis, although there are studies that emphasize the importance of physical performance status on survival in RCC patients who undergo orthopedic surgery [5]. Future studies should prospectively and systematically evaluate functional outcome and performance status in order to assess which patient is the appropriate candidate for more invasive procedures.

Additionally, while we were able to identify several risk factors for revision surgery, this study is potentially affected by sparse data bias, reflected by the large confidence intervals in some of our analyses. However, as the investigated risk factors are common in clinical practice and the potential impact on the affected patient may guide the choice of treatment, we believe that it is important to consider them. Furthermore, there are limited numbers of certain anatomic locations that were operated on and a limited number of certain risk factors or events. This limits the application of our findings to every anatomical location, and there might be risk factors for the different modes of complication that are clinically important but could not be identified with the numbers we had. However, this rather reflects the anatomic distribution of RCC metastases, the limited number of patients that undergo surgery using a megaprosthesis and the limited prevalence of certain potential risk factors. However, considering that this is the first study applying a competing risk analysis, we believe that the risk factors identified can still be useful in order to guide surgical management.

Lastly, the analysis of overall survival in patients with a metastasized carcinoma is difficult because multiple factors affect a patient’s prognosis [5,30]. While we analyzed factors that were available to us, we did not analyze specific systemic oncological treatment as we lacked comprehensive data on this subject and treatment can be expected to vary greatly depending on several factors and guidelines that were not reviewed as part of the study. However, while it is acknowledged that modern targeted treatments or immunotherapy improve patient survival in patients with a metastasized RCC, it appears difficult to identify an effect that the respective reconstruction has on survival [28], as multiple factors have to be considered. As sunitinib, the first tyrosin kinase inhibitor in the treatment of RCC, was approved in 2006 and therefore will most likely have been used more frequently in patients treated after 2006, we compared overall survival in patients undergoing megaprosthetic reconstruction prior to 2006 and in the following years, and found no difference in survival with the information available to us, although this issue should be addressed in future studies.

We found an overall survival of 29% at five years in this patient cohort, with patients with visceral metastases displaying a poorer overall survival probability. This is comparable to a study on 135 megaprostheses following an RCC metastasectomy over a 35-year study period by Hwang et al. [20] showing a five-year survival probability of 28% and patients with visceral metastases being at an increased risk for death. Potential sources of bias notwithstanding, it therefore appears that selected patients who undergo surgery for a RCC bone metastasis may have a good long-term prognosis, even in historic patient cohorts, which required surgeons to utilize a durable reconstruction method. Furthermore, when the surgical treatment of RCC metastasis is discussed, one needs to discuss the potential positive effect of metastasectomy on overall survival. There are several studies that indicate that (bone) metastasectomy, even in the case of multiple metastases, may be associated with a prolonged overall survival [4,5,10,15,31]. Considering that resection is likely to result in a segmental bone defect that can be addressed by megaprosthetic reconstruction, this aspect of the surgical approach for patients with RCC metastases might gain further importance. While the general notion of improving patient survival through metastasectomy appears attractive, one needs to acknowledge that there are no prospective, comparative studies on this issue and that previous studies acknowledge bias regarding patient selection, lacking the evaluation of other prognostic, oncological factors. Given that our study is of retrospective design and lacks a control group, we can only acknowledge the same limitations; however, the overall survival in our cohort appears favorable [15] and we currently recommend complete resection and megaprosthetic reconstruction for all patients with solitary metastases who are candidates for surgery in general.

Despite the potential advantages of resection and megaprosthetic reconstruction, we found an overall probability of revision surgery of 19%, with 7% of patients requiring exchange of the implant itself. The main concern when performing megaprosthetic reconstruction is the high long-term risk of revision surgery in up to 50% of patients [19,24]. While the risk of implant complications following reconstruction for bone metastases has previously been of less importance considering the short expected survival [7], it gains importance in patients with RCC metastases considering the long-term survival probability encountered in selected patients. A comparable and even lower probability of complications for patients undergoing megaprosthetic reconstruction for RCC metastases has been reported by other authors, despite the fact that the respective authors used a Kaplan–Meier approach for survival analysis [14,20] which would be expected to yield a higher complication rate. Furthermore, Janssen et al. [32,33], in a study on 417 femoral metastases of various origins, found that the overall complication rates between intramedullary nailing and prosthetic reconstruction are similar, but the types of complications vary, with prosthetic reconstruction having few structural complications. Nonetheless, the authors acknowledge a certain heterogeneity, with the indication that intramedullary nailing may include patients with non-RCC etiologies and poor prognosis [7,34]. On the other hand, their rate of infective complications in 9% of cases was higher for prostheses, a finding which could not be replicated in the present study. Still, there was an 18% risk of revision surgery at five years, and one in four of the revisions resulted in re-revision, which must be weighed considering the overall life expectancy and potential advantages of megaprosthetic surgery.

When investigating risk factors for revision surgery, we found that patients who underwent postoperative radiation treatment or the reconstruction of an entire bone were at increased risk for revision. The role of radiation treatment in the presence of a megaprosthesis is discussed controversially. A reduction in the risk for local recurrence has been reported [14,35,36], while on the other hand, the probability of infection in patients undergoing surgery for sarcoma has been found to be increasingly independent of the timing of radiation treatment [36]. Furthermore, one study has reported a higher risk for revision surgery following megaprosthetic reconstruction in patients with preoperative radiation treatment when combined with intralesional previous surgery [14]. Contrary to that finding, postoperative radiation treatment was a risk factor in the present study while preoperative radiation was not. However, our study is different from the previous study [14] as it includes a higher percentage of patients (41% vs. 28%) with postoperative radiation treatment, but also a higher percentage of preoperative radiation (24% vs. 19%) and higher percentage of patients with previous surgeries (29% vs. 11%). Additionally, the aforementioned cohort comprised a quarter of non-megaprosthetic reconstructions; therefore, the impact of the timing of radiation treatment and previous surgeries on the survival of megaprostheses in RCC metastases remains unclear. Another risk factor for revision surgery in our cohort was the extent of reconstruction with a longer implant and total bone replacements at increased revision risk. All total bone replacements in the current cohort required revision surgery postoperatively, but the patients ultimately survived for 15–36 months without further revisions, with one patient still alive at last follow- up. The appropriateness of total bone replacement for RCC bone metastases has been investigated by Sevelda et al. [37] in two total femoral replacements for RCC metastases, with one patient dying due to disease progression within two weeks after surgery and the other implant requiring revision for infection. This led the authors to the conclusion that total femoral replacement is rarely useful in patients with metastasized carcinoma due to the complicated recovery and high revision rate. On the other hand, total humerus reconstruction in patients with RCC metastases has been investigated by Kotwal et al. [38] in seven patients, reporting only two revision surgeries for infection and dislocation in their cohort, concluding that total humerus replacement may be an adequate treatment option in RCC metastases of the humerus. While the overall number of available cases is limited, total humerus replacement in patients with RCC appears to be associated with a more favorable implant survival, while the risks and benefits of total femoral replacement must be discussed critically with the patient where palliative care might be more appropriate in some cases [37].

## 5. Conclusions

In conclusion, selected patients who undergo megaprosthetic reconstruction following the resection of RCC metastases, even in historic patient cohorts, may achieve long-term overall survival, necessitating the use of a lasting reconstruction. Modular megaprostheses demonstrated a fairly low risk of implant revision, although postoperative radiation therapy and total bone replacements appear to be associated with an increased risk.

## Figures and Tables

**Table 1:** Patient demographics and details on surgical and oncological factors.

Variable	% (n)
Male	71 (61/86)
Smokers	14 (12/86)
Diabetes	12 (10/86)
Pathologic fracture	54 (46/86)
Previous non-megaprosthetic reconstruction	29 (25/86)
Intramedullary nail	9 (8/25)
Plate or screw fixation	14 (12/25)
Arthroplasty	6 (5/25)
Resection margin	
Tumor-free margins	78 (67/86)
Intralesional margin	22 (19/86)
Postoperative local radiation	41 (35/86)
Preoperative local radiation	24 (21/86)
Systemic therapy for primary tumor	37 (32/86)
Surgery for renal tumor	88 (76/86)
Visceral metastasis at surgery	21 (18/86)
Pulmonary metastasis at surgery	41 (36/86)
Solitary bone metastasis	31 (27/86)
Time of metastasis	
Synchronous	28 (24/86)
Metachronous early (<2 years from the initial diagnosis)	17 (15/86)
Metachronous late (>2 years)	55 (47/86)

**Table 2:** Patient, surgical and oncological details.

Variable	Mean (±SD)
Body mass index (BMI in kg/m^2^)	29 (7.1)
Age at initial surgery in years	69 (6.8)
Reconstruction length in mm	233 (64)
Follow-up in months	53 (29.5)
Time from initial diagnosis to implantation of a megaprosthesis	84 (39.8)

**Table 3:** Details on prosthetic reconstruction.

Variable	% (n)
**Prosthetic location**	
Proximal femur (PFR)	38 (33/86)
Distal femur (DFR)	23 (20/86)
Proximal humerus (PHR)	23 (20/86)
Intercalary (IP)	9 (8/86)
Total humerus (THR)	2 (2/86)
Proximal tibial replacement (PTR)	1 (1/86)
Total femoral (TFR)	1 (1/86)
Distal humerus (DHR)	1 (1/86)

**Table 4:** Risk factors for revision surgery.

Variable	Hazard Ratio	95% Confidence Interval	*p*-Value
Diabetes	1.01	0.237–4.309	0.99
Smoking	0.40	0.055–2.971	0.37
Age at surgery	0.99	0.960–1.031	0.77
Pathological fracture	1.17	0.450–3.045	0.75
Previous non-megaprosthetic surgery	1.55	0.575–4.186	0.39
Cemented stem	0.49	0.151–1.577	0.23
Blood transfusion	2.06	0.789–5.352	0.14

## Data Availability

Raw data is available upon reasonable request.
